# Supplementary material for: Sialyl Lewisx-P-selectin cascade mediates tumor–mesothelial adhesion in ascitic fluid shear flow
Source: Nat Commun. 2019 Jun 3;10:2406. doi: 10.1038/s41467-019-10334-6 (PMC6547673; doi:10.1038/s41467-019-10334-6)
Supplement: Supplementary file 1 — Supplementary Information [file 41467_2019_10334_MOESM1_ESM.pdf]

Sialyl Lewis^{x}-P-selectin Cascade Mediates Tumor-Mesothelial Adhesion in Ascitic Fluid Shear Flow

Shan-Shan Li et al.

Supplementary Information

SUPPLEMENTARY METHODS

Kaplan-Meier analysis

Progression-free survival (PFS) analysis was performed in an integrative ovarian cancer datasets[1]. Patients with advanced-stage (FIGO stage III-IV) ovarian cancer (all clinical subtypes) were divided into low- and high-expression groups according to the mRNA level of specific genes. The risk differences of two groups were estimated using the log-rank test. The cutoff point giving the most pronounced *P* value was selected.

Supplementary Table 1

Antibodies and recombinant proteins

Antibody	Catalog no., source	Concentration
Anti-β-actin	A5060, Sigma-Aldrich	1:5000
Anti-CA19-9	ab15146, Abcam	1:100
HECA-452	550407, BD Biosciences	FC:10 μg mL^{-1}, WB:1:50
CSLEX-1	551344, BD Biosciences	10 μg mL^{-1}
Anti-IGF-1R β	3027, Cell Signaling	1:1000
Anti-IGF-1R α (N-20)	SC-712, Santa Cruz Biotechnology	1:1000
Anti-phospho-IGF-1R β	3024s, Cell Signaling	1:1000
Anti-E-selectin	555648, BD Biosciences	20 μg mL^{-1}
Anti-L-selectin	555522, BD Biosciences	20 μg mL^{-1}
Anti-P-selectin	555542, BD Biosciences	20 μg mL^{-1}
Alexa Fluor 488 goat anti-rat IgM	A-21212, Life technology	1:500
Alexa Fluor 488 goat anti-mouse IgG, IgM	A-10667, Life technology	1:500
Alexa Fluor 488 goat anti-human IgG	A-11013, Life technology	1:500
Mouse IgG	I5381, Sigma-Aldrich	Same as target antibodies
Mouse IgM	555581, BD Biosciences	Same as target antibodies
Goat anti-Rat IgM Secondary Antibody, HRP	31476, Invitrogen	1:100000
Goat anti-mouse IgG (H+L)-HRP conjugated	170-6516, Bio-Rad	1:3000
Goat anti-rabbit IgG (H+L)-HRP conjugated	170-6515, Bio-Rad	1:2500

Recombinant proteins

Recombinant protein	Catalog no., source	Working concentration
Recombinant Human E-Selectin Fc Chimera	724-ES, R&D systems	1~5 μg mL^{-1}
Recombinant Human P-Selectin Fc Chimera	137-PS, R&D systems	1~5 μg mL^{-1}
Recombinant Human L-Selectin Fc Chimera	728-LS, R&D systems	1~5 μg mL^{-1}
Recombinant Human IgG1 Fc	110-HG, R&D systems	Same as selectin-Fc
Human Fibronectin	354008, Corning	10 μg mL^{-1}

Supplementary Table 2

Biochemical inhibitors

Inhibitor	Description	Catalog no., source	Concentration
Genistein	Protein tyrosine kinase inhibitor	345834, Calbiochem	50 µM
AG1024	IGF-1R inhibitor	121767, Calbiochem	10 µM
AG1478	EGFR inhibitor	65855, Calbiochem	200 nM
K252a	Met inhibitor	420298, Calbiochem	50 nM
SU5402	FGF-1R inhibitor	572630, Calbiochem	50 µM

Supplementary Table 3

DNA primers

Gene	Forward 5' – 3'	Reverse 5' – 3'
Primers for quantitative real-time PCR:		
E-selectin	CAGCAAAGGTACACACACCTG	CAGACCCACACATTGTTGACTT
P-selectin	TCCTCACAGCCACCTAGGAA	GGAAACAGGGTTGGTCCAGA
L-selectin	TCTGTTACACAGCTTCTTGCCA	GGCCCATAGTACCCCACATC
B4GalT4	GTGGGCCACCAGTAACTACTT	TCGTGGATGCTTCATTAGTCAGA
ST3Gal3	GCCTGCTGAATTAGCCACCAA	GCCCACTTGCGAAAGGAGT
ST3Gal4	CCACTTCGACCCCAAAGTAGA	CGCACCCGCTTCTTATCACT
FUT5	GAGGGCGAAGGGTATGTGTG	GCATCGCAACACATCCACAG
GAPDH	GGAGCGAGATCCCTCCAAAAT	GGCTGTTGTCATACTTCTCATGG
CD24	CTCCTACCCACGCAGATTTATTC	AGAGTGAGACCACGAAGAGAC
IGF-1R	TGTCCAGGCCAAAACAGGA	CAACCCTCCCACGATCAACA
N-cadherin	AATCGTGTCTCAGGCTCCAA	TGGGATTGCCTTCCATGTCT
Vimentin	CCCTCACCTGTGAAGTGGAT	TGACGAGCCATTTCCTCCTT

Gene	Forward 5' – 3'	Reverse 5' – 3'
Primers for genotyping:		
Selp^{WT}	TTGTAAATCAGAAGGAAGTGG	AGAGTTACTCTTGATGTAGATCTCC
Selp^{Mutant}	CTGAATGAACTGCAGGACGA	ATACTTTCTCGGCAGGAGCA
Rag2^{WT}	ATCAATGGTTCACCCCTTTG	TCATGTGAAAGCAGTTCAGGAC
Rag2^{Mutant}	CCGCCATATGCATCCAAC	CAGCGCTCCTCCTGATACTC

Gene target	Sequence 5' – 3'
shRNA for knockdown:	
FUT5	CCGGCACTGCCGACTCCAGTGTGTACTCGAGTACACACTGGAGTCGGCAGTGTTTTTG

SUPPLEMENTARY FIGURES

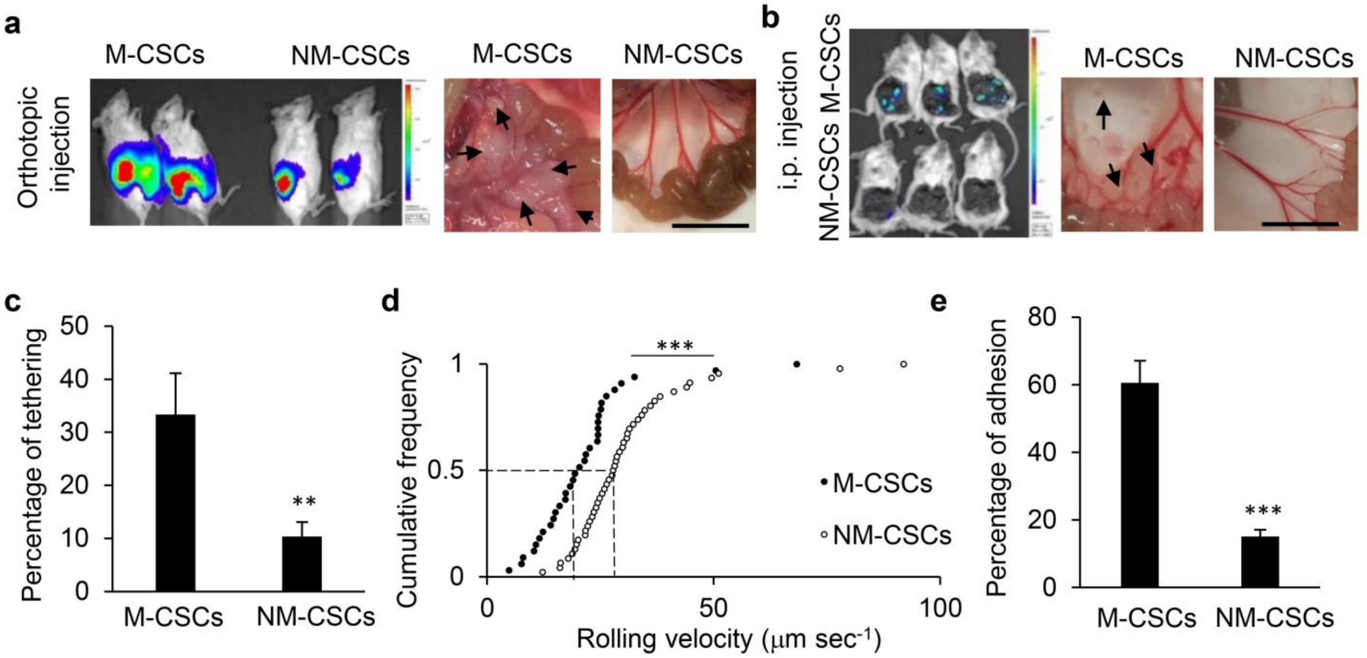

Supplementary Figure 1 Characterization of HEYA8 M-CSCs. NOD/SCID mice engrafted with HEYA8 M-CSCs or NM-CSCs via orthotopic (**a**) or i.p. (**b**) injection. N = 3 mice per group, *In vivo* tumor xenograft experiments were conducted twice. Left: representative bioluminescence image. Right: representative views of metastases in the peritoneal cavity. Scale bar, 1cm. **c** Percentage of tethering of HEYA8 M-CSCs and NM-CSCs on HPMCs at 0.05 dyne cm^{-2}. **d** Cancer spheroids rolling velocities on HPMCs at 0.05 dyne cm^{-2}. **e** Percentage of HEYA8 M-CSCs and NM-CSCs adhered onto HPMCs. n = 31/45. Data (mean ± SEM) from one of three independent experiments. Statistical analysis using chi-squared test (**c**, **e**) and one-tailed unpaired Student's *t* test (**d**). **, $P < 0.01$; ***, $P < 0.001$.

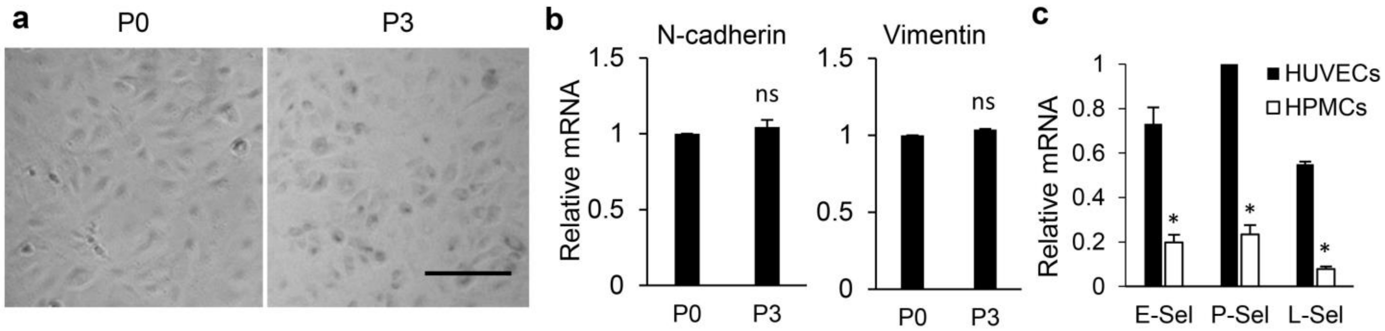

Supplementary Figure 2 Morphology and gene expression of HPMCs. **a** Representative brightfield images of HPMCs at low passages (passage 0 and 3). Scale bar, 100 μm. **b** The mRNA expression of EMT markers in different passages of HPMCs. **c** The mRNA expression of selectins in HPMCs and HUVECs. Data (mean ± SEM) from two biological replicates, n = 2, paired (**b**) or unpaired (**c**) Student's *t* test. ns, not significant; *, $p < 0.05$.

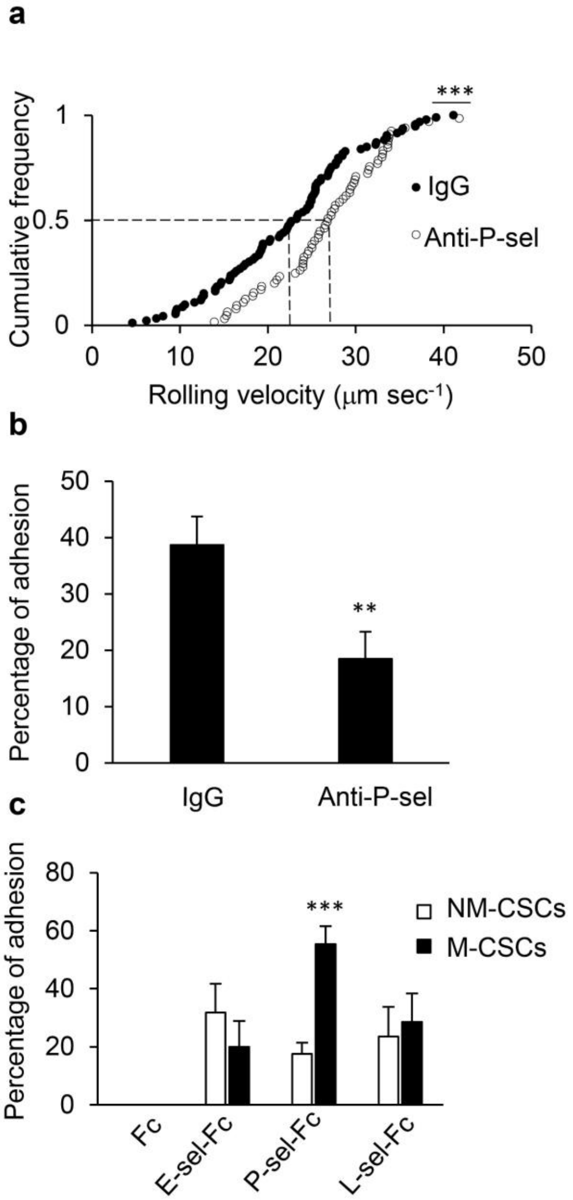

Supplementary Figure 3 P-selectins mediates HEYA8 M-CSC-HPMC interaction. **a**, **b** Cumulative frequency (**a**) and adhesion percentage (**b**) of HEYA8 M-CSCs on HPMCs blocked with anti-P-selectin at 0.05 dyne cm^{-2}. n = 93/65. **c** Percentage of HEYA8 M-CSCs and NM-CSCs adhered onto selectins at 0.05 dyne cm^{-2}. n = 36/76, 20/22, 65/97, 21/17. Data (mean ± SEM) from one of three independent experiments. Statistical analysis using unpaired Student's *t* test (**a**) and chi-squared test (**b**, **c**). **, $p < 0.01$; ***, $p < 0.001$.

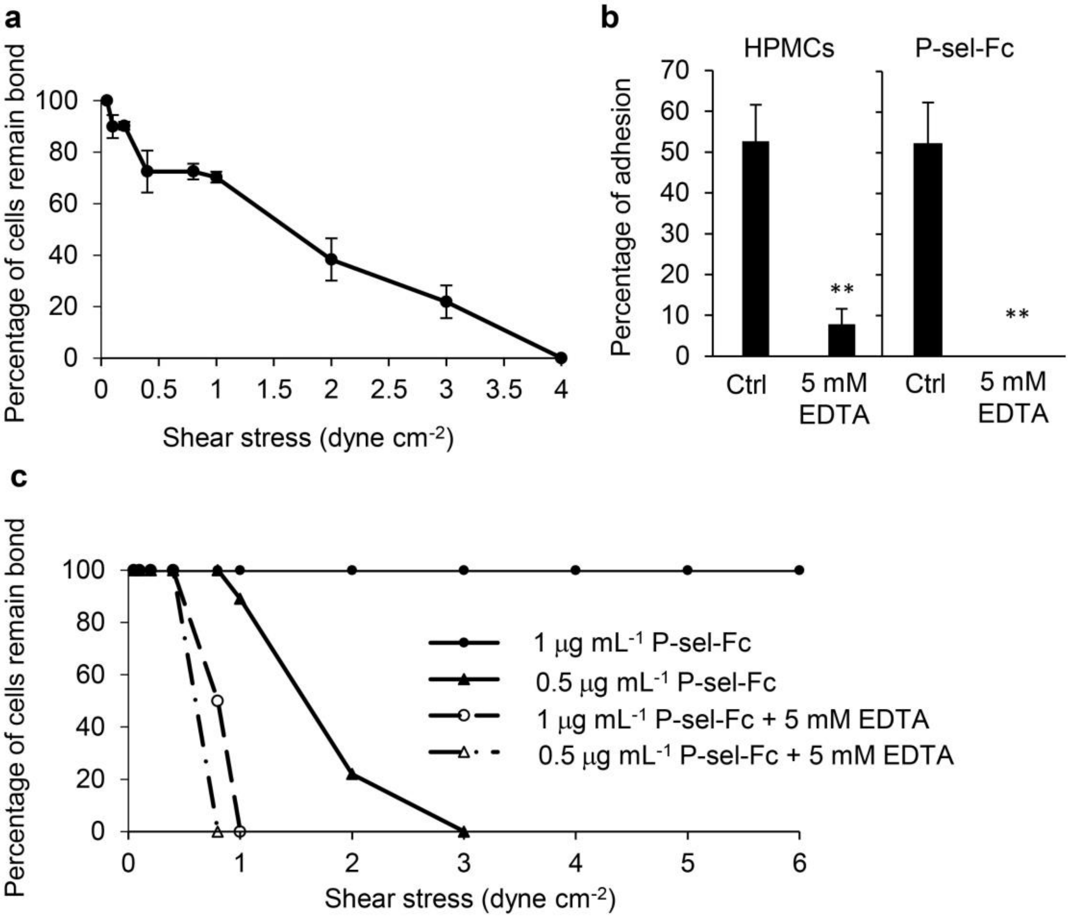

Supplementary Figure 4 The P-selectin-mediated binding is shear-resistant and Ca^{2+}-dependent. **a** Detachment of SKOV-3 M-CSCs on HPMCs. **b** Percentage of SKOV-3 M-CSCs adhered on HPMCs or P-selectin-Fc in the presence or absence of in EDTA at 0.05 dyne cm^{-2}. n = 31/58, 21/11. Data (mean ± SEM) from one of two independent experiments, chi-square test. **, $p < 0.01$. **c** Detachment of SKOV-3 M-CSCs on P-selectin-Fc the presence or absence of EDTA.

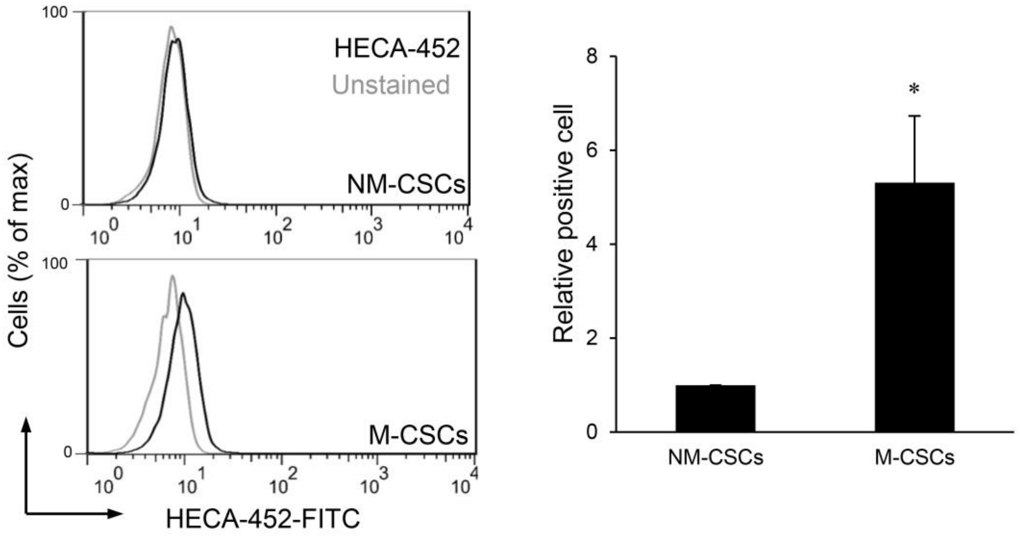

Supplementary Figure 5 sLe$^{a/x}$ expression on HEYA8 M-CSCs and NM-CSCs cells. Left: representative images of flowcytometry. Right: relative positive cell plot. Data (mean ± SEM) from three biological replicates, n = 3, unpaired Student *t* test. *, $p < 0.05$.

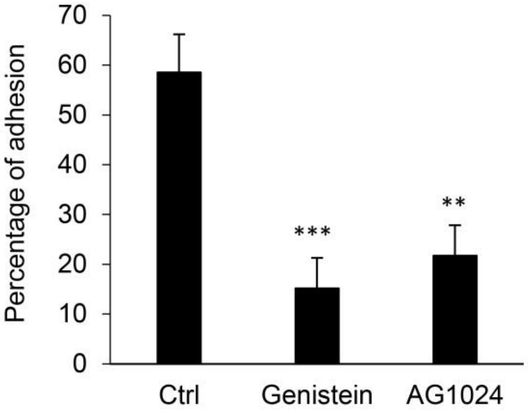

Supplementary Figure 6 Percentage of adherent M-CSCs on P-selectin-Fc after inhibitor treatment. SKOV-3 M-CSCs were treated with or without general RTK inhibitors genistein or IGF-1R inhibitor AG1024 before perfusion on P-selectin-Fc recombinant protein. n = 41, 33, 46. Data (mean ± SEM) from one of three independent experiments, chi-square test. **, $P < 0.01$; ***, $P < 0.001$.

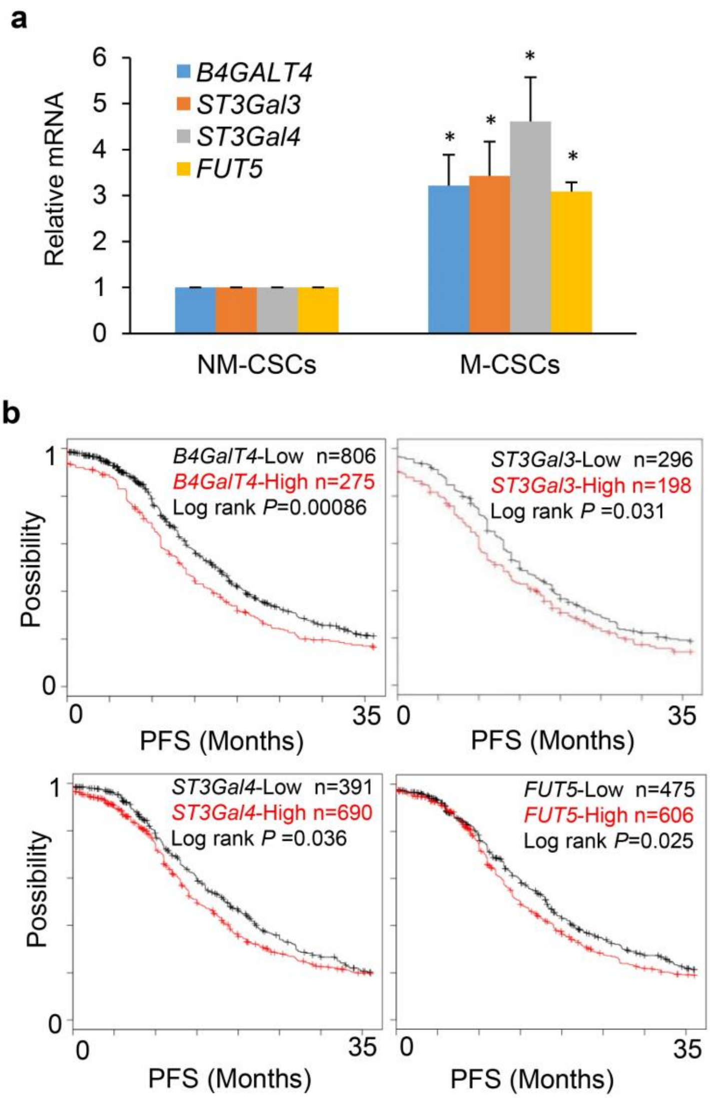

Supplementary Figure 7 The correlation between glycosyltransferases with metastatic properties and patient prognosis. **a** The mRNA expression of glycogenes in HEYA8 M-CSCs and NM-CSCs. Data (mean ± SEM) from three biological replicates, n = 3, unpaired Student *t* test. *, P < 0.05. **b** Kaplan-Meier progression-free survival (PFS) analysis of *B4GalT4*, *ST3Gal3*, *ST3Gal4* and *FUT5* expression in advanced-stage ovarian cancer patients with high (red) and low (black) expression levels of specific genes. Statistical analysis using log-rank test.

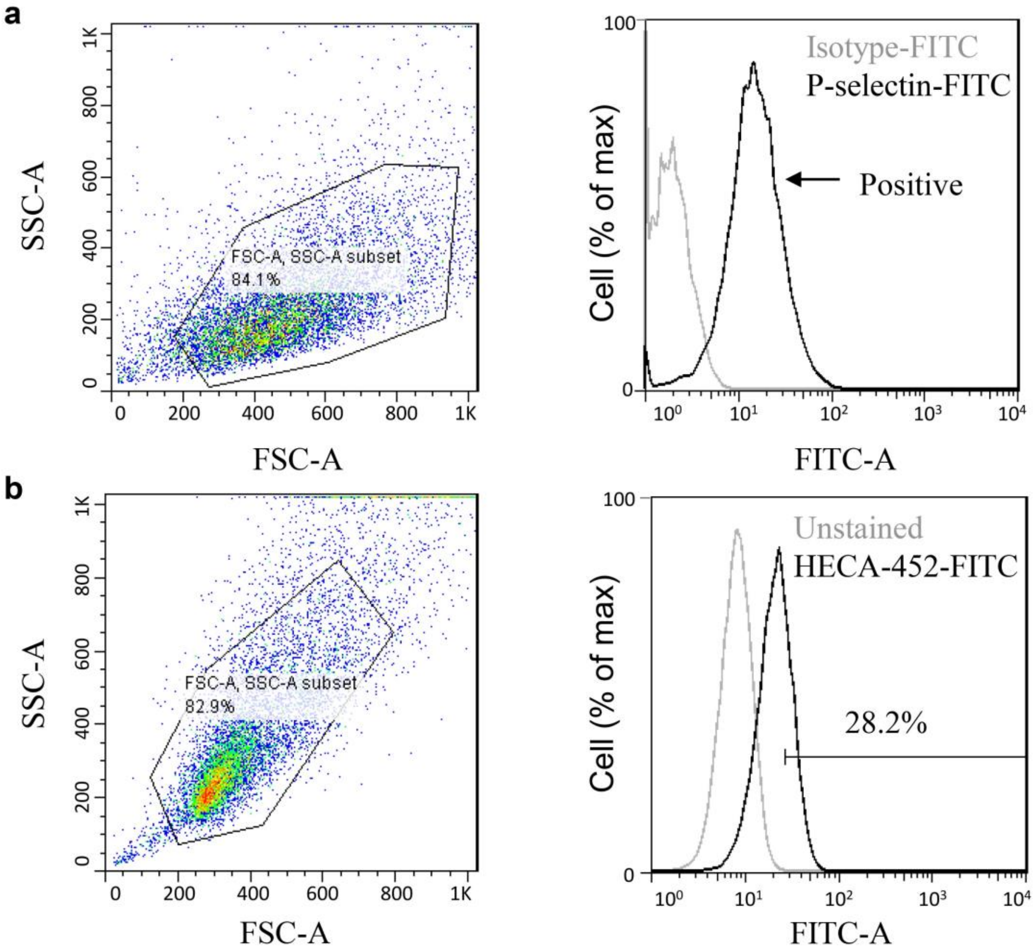

Supplementary Figure 8 Gating strategies for flow cytometry analysis. **a** Representative gating strategy for cell surface staining of selectin on HPMCs. **b** Representative gating strategy for cell surface staining of HECA-452 on SKOV-3 M-CSCs.

Figure 5c

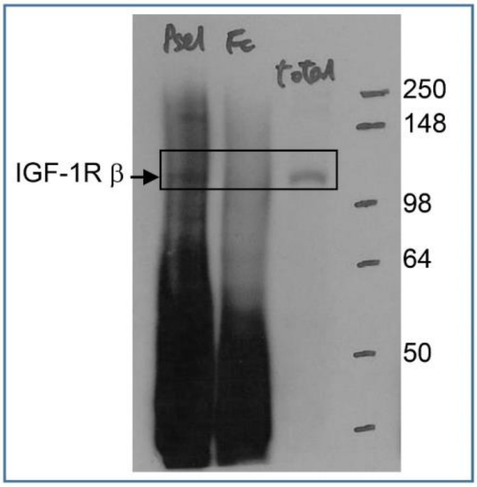

Figure 5d

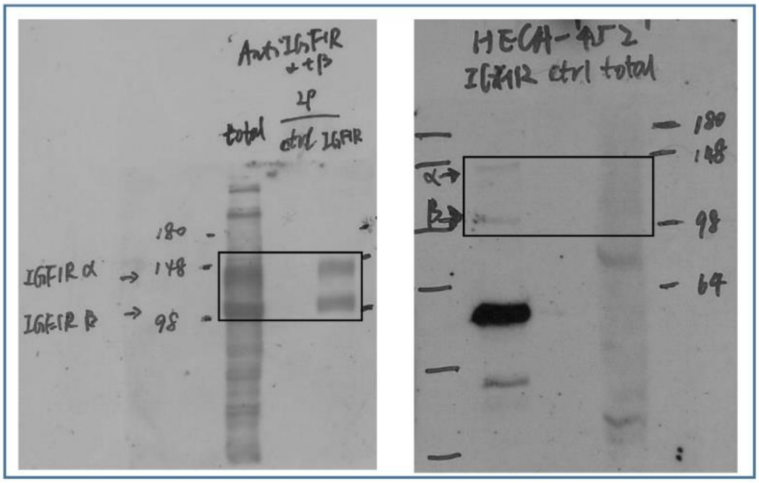

Figure 5e

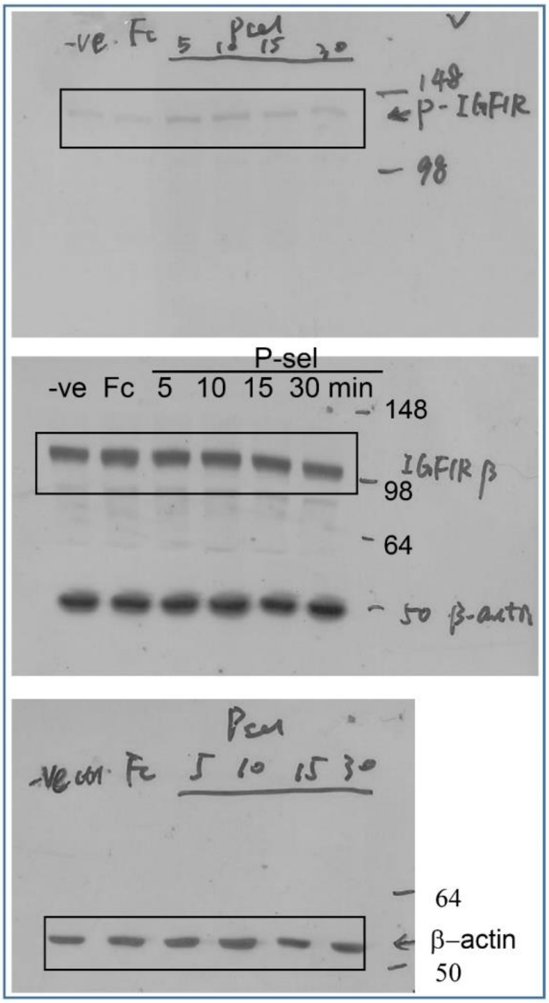

Supplementary Figure 9 Full blots shown in Figure 5c-5e.

Supplementary Table 4 Metastasis in $Selp^{WT}$ and $Selp^{-/-}$ mice

	$Selp^{WT}$	$Selp^{-/-}$
Metastases (no.)	45 ± 6	11 ± 2 (orthotopic) *
	103 ± 6	47 ± 3 (i.p.) **
Ascites volume (mL)	0.5 ± 0.06	0.1 ± 0.02 (orthotopic) **
	0.6 ± 0.07	0.2 ± 0.02 (i.p.) *

Results are represented with mean ± SEM. n = 6 mice (orthotopic) or 9 mice (i.p.) from two independent experiments. Statistical analysis using unpaired Student's *t* test. ns, not significant. *, $P < 0.05$; **, $P < 0.01$.

Supplementary Table 5 Metastasis in mice with NS or FUT5 shRNA transduced M-CSCs

	NS shRNA	FUT5 shRNA
Metastases (no.)	24 ± 6	4 ± 3 (orthotopic) *
	40 ± 9	13 ± 5 (i.p.) *
Ascites volume (mL)	0.9 ± 0.26	0.1 ± 0.07 (orthotopic) *
	0.6 ± 0.02	0.2 ± 0.07 (i.p.) *

Results are represented with mean ± SEM. n = 3 mice (orthotopic) or 6 mice (i.p.) from two independent experiments. Statistical analysis using unpaired Student's *t* test. ns, not significant. *, $P < 0.05$.

SUPPLEMENTARY REFERENCES

1. Gyorffy, B., Lanczky, A. & Szallasi, Z. Implementing an online tool for genome-wide validation of survival-associated biomarkers in ovarian-cancer using microarray data from 1287 patients. *Endocr Relat Cancer* **19**, 197-208 (2012).
