# Supplementary material for: Sialyl Lewisx-P-selectin cascade mediates tumor–mesothelial adhesion in ascitic fluid shear flow
Source: Nat Commun. 2019 Jun 3;10:2406. doi: 10.1038/s41467-019-10334-6 (PMC6547673; doi:10.1038/s41467-019-10334-6)
Supplement: Supplementary file 2 — Description of Additional Supplementary Files [file 41467_2019_10334_MOESM2_ESM.docx]

**Description of Supplementary Files**

**File Name:** Supplementary Movie 1

**Description:** Perfusion of fluorescently labeled M-CSCs on HPMCs pretreated under 0.05 dynes cm-2.

**File Name:** Supplementary Movie 2

**Description:** Perfusion of fluorescently labeled M-CSCs on HPMCs blocked with anti-P-selectin under 0.05 dynes cm-2.

**File Name:** Supplementary Movie 3

**Description:** Perfusion of fluorescently labeled ascitic tumor spheroids derived from a patient with ovarian cancer on Fc under 0.05 dynes cm-2.

**File Name:** Supplementary Movie 4

**Description:** Perfusion of fluorescently labeled ascitic tumor spheroids derived from a patient with ovarian cancer (the same patient sample as Extended Data Video 3) on P-selectin-Fc under 0.05 dynes cm-2.
