# Supplementary material for: Sialyl Lewisx-P-selectin cascade mediates tumor–mesothelial adhesion in ascitic fluid shear flow
Source: Nat Commun. 2019 Jun 3;10:2406. doi: 10.1038/s41467-019-10334-6 (PMC6547673; doi:10.1038/s41467-019-10334-6)
Supplement: Supplementary file 7 — Reporting Summary [file 41467_2019_10334_MOESM7_ESM.pdf]

naturerearch

Corresponding author(s): DBPR NCOMMS-18-09972A

Last updated by author(s): Apr 11, 2019

# Reporting Summary

Nature Research wishes to improve the reproducibility of the work that we publish. This form provides structure for consistency and transparency in reporting. For further information on Nature Research policies, see Authors & Referees and the Editorial Policy Checklist.

## Statistics

For all statistical analyses, confirm that the following items are present in the figure legend, table legend, main text, or Methods section.

| n/a | Confirmed | |
|---|---|---|
| ☐ | ☒ | The exact sample size (*n*) for each experimental group/condition, given as a discrete number and unit of measurement |
| ☐ | ☒ | A statement on whether measurements were taken from distinct samples or whether the same sample was measured repeatedly |
| ☐ | ☒ | The statistical test(s) used AND whether they are one- or two-sided<br>*Only common tests should be described solely by name; describe more complex techniques in the Methods section.* |
| ☒ | ☐ | A description of all covariates tested |
| ☒ | ☐ | A description of any assumptions or corrections, such as tests of normality and adjustment for multiple comparisons |
| ☐ | ☒ | A full description of the statistical parameters including central tendency (e.g. means) or other basic estimates (e.g. regression coefficient) AND variation (e.g. standard deviation) or associated estimates of uncertainty (e.g. confidence intervals) |
| ☒ | ☐ | For null hypothesis testing, the test statistic (e.g. *F*, *t*, *r*) with confidence intervals, effect sizes, degrees of freedom and *P* value noted<br>*Give P values as exact values whenever suitable.* |
| ☒ | ☐ | For Bayesian analysis, information on the choice of priors and Markov chain Monte Carlo settings |
| ☒ | ☐ | For hierarchical and complex designs, identification of the appropriate level for tests and full reporting of outcomes |
| ☒ | ☐ | Estimates of effect sizes (e.g. Cohen's *d*, Pearson's *r*), indicating how they were calculated |

*Our web collection on statistics for biologists contains articles on many of the points above.*

## Software and code

Policy information about availability of computer code

| | |
|---|---|
| Data collection | *Provide a description of all commercial, open source and custom code used to collect the data in this study, specifying the version used OR state that no software was used.* |
| Data analysis | *Provide a description of all commercial, open source and custom code used to analyse the data in this study, specifying the version used OR state that no software was used.* |

For manuscripts utilizing custom algorithms or software that are central to the research but not yet described in published literature, software must be made available to editors/reviewers. We strongly encourage code deposition in a community repository (e.g. GitHub). See the Nature Research guidelines for submitting code & software for further information.

## Data

Policy information about availability of data

All manuscripts must include a data availability statement. This statement should provide the following information, where applicable:

- Accession codes, unique identifiers, or web links for publicly available datasets
- A list of figures that have associated raw data
- A description of any restrictions on data availability

The RNA sequencing data have been deposited in the NCBI Trace Archive under the accession code PRJNA530706. All the other data supporting the findings of this study are available within the article and its supplementary information files or from the corresponding author upon reasonable request.

# Field-specific reporting

Please select the one below that is the best fit for your research. If you are not sure, read the appropriate sections before making your selection.

☐ Life sciences ☐ Behavioural & social sciences ☐ Ecological, evolutionary & environmental sciences

For a reference copy of the document with all sections, see nature.com/documents/nr-reporting-summary-flat.pdf

# Life sciences study design

All studies must disclose on these points even when the disclosure is negative.

| | |
|---|---|
| Sample size | n = the number of biological replicates used in each experimental group. |
| Data exclusions | No data exclusion. |
| Replication | Experiments are conducted two or three times independently using distinct samples. All attempts at replication were successful. |
| Randomization | Samples were randomly allocated into experimental groups. |
| Blinding | Investigators are not blinded to group allocation during data collection and analysis. |

# Behavioural & social sciences study design

All studies must disclose on these points even when the disclosure is negative.

| | |
|---|---|
| Study description | *Briefly describe the study type including whether data are quantitative, qualitative, or mixed-methods (e.g. qualitative cross-sectional, quantitative experimental, mixed-methods case study).* |
| Research sample | *State the research sample (e.g. Harvard university undergraduates, villagers in rural India) and provide relevant demographic information (e.g. age, sex) and indicate whether the sample is representative. Provide a rationale for the study sample chosen. For studies involving existing datasets, please describe the dataset and source.* |
| Sampling strategy | *Describe the sampling procedure (e.g. random, snowball, stratified, convenience). Describe the statistical methods that were used to predetermine sample size OR if no sample-size calculation was performed, describe how sample sizes were chosen and provide a rationale for why these sample sizes are sufficient. For qualitative data, please indicate whether data saturation was considered, and what criteria were used to decide that no further sampling was needed.* |
| Data collection | *Provide details about the data collection procedure, including the instruments or devices used to record the data (e.g. pen and paper, computer, eye tracker, video or audio equipment) whether anyone was present besides the participant(s) and the researcher, and whether the researcher was blind to experimental condition and/or the study hypothesis during data collection.* |
| Timing | *Indicate the start and stop dates of data collection. If there is a gap between collection periods, state the dates for each sample cohort.* |
| Data exclusions | *If no data were excluded from the analyses, state so OR if data were excluded, provide the exact number of exclusions and the rationale behind them, indicating whether exclusion criteria were pre-established.* |
| Non-participation | *State how many participants dropped out/declined participation and the reason(s) given OR provide response rate OR state that no participants dropped out/declined participation.* |
| Randomization | *If participants were not allocated into experimental groups, state so OR describe how participants were allocated to groups, and if allocation was not random, describe how covariates were controlled.* |

# Ecological, evolutionary & environmental sciences study design

All studies must disclose on these points even when the disclosure is negative.

| | |
|---|---|
| Study description | *Briefly describe the study. For quantitative data include treatment factors and interactions, design structure (e.g. factorial, nested, hierarchical), nature and number of experimental units and replicates.* |
| Research sample | *Describe the research sample (e.g. a group of tagged Passer domesticus, all Stenocereus thurberi within Organ Pipe Cactus National Monument), and provide a rationale for the sample choice. When relevant, describe the organism taxa, source, sex, age range and any manipulations. State what population the sample is meant to represent when applicable. For studies involving existing datasets, describe the data and its source.* |

| | |
|---|---|
| Sampling strategy | *Note the sampling procedure. Describe the statistical methods that were used to predetermine sample size OR if no sample-size calculation was performed, describe how sample sizes were chosen and provide a rationale for why these sample sizes are sufficient.* |
| Data collection | *Describe the data collection procedure, including who recorded the data and how.* |
| Timing and spatial scale | *Indicate the start and stop dates of data collection, noting the frequency and periodicity of sampling and providing a rationale for these choices. If there is a gap between collection periods, state the dates for each sample cohort. Specify the spatial scale from which the data are taken* |
| Data exclusions | *If no data were excluded from the analyses, state so OR if data were excluded, describe the exclusions and the rationale behind them, indicating whether exclusion criteria were pre-established.* |
| Reproducibility | *Describe the measures taken to verify the reproducibility of experimental findings. For each experiment, note whether any attempts to repeat the experiment failed OR state that all attempts to repeat the experiment were successful.* |
| Randomization | *Describe how samples/organisms/participants were allocated into groups. If allocation was not random, describe how covariates were controlled. If this is not relevant to your study, explain why.* |
| Blinding | *Describe the extent of blinding used during data acquisition and analysis. If blinding was not possible, describe why OR explain why blinding was not relevant to your study.* |

Did the study involve field work? ☐ Yes ☐ No

## Field work, collection and transport

| | |
|---|---|
| Field conditions | *Describe the study conditions for field work, providing relevant parameters (e.g. temperature, rainfall).* |
| Location | *State the location of the sampling or experiment, providing relevant parameters (e.g. latitude and longitude, elevation, water depth).* |
| Access and import/export | *Describe the efforts you have made to access habitats and to collect and import/export your samples in a responsible manner and in compliance with local, national and international laws, noting any permits that were obtained (give the name of the issuing authority, the date of issue, and any identifying information).* |
| Disturbance | *Describe any disturbance caused by the study and how it was minimized.* |

# Reporting for specific materials, systems and methods

We require information from authors about some types of materials, experimental systems and methods used in many studies. Here, indicate whether each material, system or method listed is relevant to your study. If you are not sure if a list item applies to your research, read the appropriate section before selecting a response.

### Materials & experimental systems

| n/a | Involved in the study | |
|---|---|---|
| ☐ | ☒ | Antibodies |
| ☐ | ☒ | Eukaryotic cell lines |
| ☒ | ☐ | Palaeontology |
| ☐ | ☒ | Animals and other organisms |
| ☒ | ☐ | Human research participants |
| ☒ | ☐ | Clinical data |

### Methods

| n/a | Involved in the study | |
|---|---|---|
| ☒ | ☐ | ChIP-seq |
| ☐ | ☒ | Flow cytometry |
| ☒ | ☐ | MRI-based neuroimaging |

## Antibodies

| | |
|---|---|
| Antibodies used | Anti-b-actin (A5060, Sigma-Aldrich), Anti-CA19-9 (ab15146, Abcam), HECA-452 (550407, BD Biosciences),CSLEX-1 (551344, BD Biosciences),Anti-IGF-1R b (3027, Cell Signaling), Anti-IGF-1R a (N-20, SC-712, Santa Cruz Biotechnology), anti-IGF-1R a (24-60, Invitrogen) Anti-phospho-IGF-1R b (3024s, Cell Signaling), Anti-E-selectin (555648, BD Biosciences), Anti-L-selectin (555522, BD Biosciences), Anti-P-selectin (555542, BD Biosciences), Alexa Fluor 488 goat anti-rat IgM (A-21212, Life technology), Alexa Fluor 488 goat anti-mouse IgG, IgM (A-10667, Life technology), Alexa Fluor 488 goat anti-human IgG (A-11013, Life technology), Mouse IgG (I5381, Sigma-Aldrich), Mouse IgM (555581, BD Biosciences), Goat anti-Rat IgM Secondary Antibody, HRP (31476, Invitrogen), Goat anti-mouse IgG (H+L)-HRP conjugated (170-6516, Bio-Rad), (170-6515, Bio-Rad). |
| Validation | Validation statements can be found on manufacturer's website. |

# Eukaryotic cell lines

Policy information about cell lines

| | |
|---|---|
| Cell line source(s) | Human ovarian cancer cell lines SKOV-3 and HEYA8 (kind gifts from Dr. N. Auersperg at University of British Columbia and Dr. J Liu at MD Anderson Cancer Center, respectively). HEK293 cells (CRL-1573, ATCC). HUVECs (CC-2519, Clonetics). |
| Authentication | SKOV-3 has been authenticated in 2014. Other cell lines are not authenticated in our lab after receiving. |
| Mycoplasma contamination | All cell lines tested negative for mycoplasma contamination several years ago. |
| Commonly misidentified lines (See ICLAC register) | No commonly misidentified lines used in this study. |

# Animals and other organisms

Policy information about studies involving animals; ARRIVE guidelines recommended for reporting animal research

| | |
|---|---|
| Laboratory animals | C57BL/6 Selp-/- (JAX #002289, The Jackson Laboratory) and Rag2-/- (JAX #008449, The Jackson Laboratory) mice. NOD.CB17-Prkdcscid/J (NOD SCID) (Laboratory Animal Unit, The University of Hong Kong). |
| Wild animals | n/a |
| Field-collected samples | n/a |
| Ethics oversight | All animal experiment protocols were approved by the Committee on the Use of Live Animals in Teaching and Research at the University of Hong Kong. |

Note that full information on the approval of the study protocol must also be provided in the manuscript.

# Flow Cytometry

## Plots

Confirm that:

☒ The axis labels state the marker and fluorochrome used (e.g. CD4-FITC).

☒ The axis scales are clearly visible. Include numbers along axes only for bottom left plot of group (a 'group' is an analysis of identical markers).

☒ All plots are contour plots with outliers or pseudocolor plots.

☒ A numerical value for number of cells or percentage (with statistics) is provided.

## Methodology

| | |
|---|---|
| Sample preparation | Cultured adherent cells or tumor spheroids were detached with 2 mM EDTA PBS, then re-suspended in PBS. Cells were incubated with primary antibodies, selectin-Fc chimeras or isotype control per the manufacturer's instructions. The primary antibody or recombinant protein were stained with appropriate Alexa Fluor 488 secondary antibodies. |
| Instrument | FACS AriaIII, BD Biosciences |
| Software | FlowJo |
| Cell population abundance | Collected 10,000 events for each sample. |
| Gating strategy | To gate out cellular debris, dot plots displaying FSC/SSC were created and cellular debris visible in the bottom left corner of the plot were excluded by the gate. Flow cytometry was conducted in the presence of isotype or unstained controls to identify positive dataset. |

☒ Tick this box to confirm that a figure exemplifying the gating strategy is provided in the Supplementary Information.
